# Prenatal alcohol exposure alters gene expression in the rat brain: Experimental design and bioinformatic analysis of microarray data

**DOI:** 10.1016/j.dib.2015.05.007

**Published:** 2015-06-09

**Authors:** Alexandre A. Lussier, Katarzyna A. Stepien, Joanne Weinberg, Michael S. Kobor

**Affiliations:** a Department of Medical Genetics, University of British Columbia, Vancouver, British Columbia, Canada; b Department of Cellular and Physiological Sciences, University of British Columbia, 2350 Health Sciences Mall, Vancouver, British Columbia, Canada V6T 1Z3; c Department of Medical Genetics, Centre for Molecular Medicine and Therapeutics, Child and Family Research Institute, Human Early Learning Partnership, University of British Columbia, Room 2024, 950 West 28th Avenue, Vancouver, British Columbia, Canada V5Z 4H4

## Abstract

We previously identified gene expression changes in the prefrontal cortex and hippocampus of rats prenatally exposed to alcohol under both steady-state and challenge conditions (*Lussier et al., 2015, Alcohol.: Clin. Exp. Res., 39, 251–261*). In this study, adult female rats from three prenatal treatment groups (ad libitum-fed control, pair-fed, and ethanol-fed) were injected with physiological saline solution or complete Freund׳s adjuvant (CFA) to induce arthritis (adjuvant-induced arthritis, AA). The prefrontal cortex and hippocampus were collected 16 days (peak of arthritis) or 39 days (during recovery) following injection, and whole genome gene expression was assayed using Illumina׳s RatRef-12 expression microarray. Here, we provide additional metadata, detailed explanations of data pre-processing steps and quality control, as well as a basic framework for the bioinformatic analyses performed. The datasets from this study are publicly available on the GEO repository (accession number GSE63561).

**Table:** 

**Specifications**	
Organism/cell line/tissue	*Rattus norvegicus* – Sprague Dawley (Charles River-derived) – prefrontal cortex and hippocampus
Sex	Female
Sequencer or array type	Illumina Rat-Ref12 beadarray
Data format	Raw and quantile normalized
Experimental factors	Prenatal treatment: control, alcohol, pairfed
	Postnatal treatment: saline-injected, adjuvant-injected
	Sample collection: day 16 post-injection (peak of arthritis), day 39 post-injection (during recovery)
Experimental features	Adult female rats from three treatment groups (prenatal alcohol exposed, pair-fed, ad libitum-fed control) were injected with saline or complete Freund׳s adjuvant. The prefrontal cortex and hippocampus of these animals were collected either 16 or 39 days following injection to test the effects of prenatal alcohol exposure and adjuvant-induced arthritis on gene expression.
Consent	NA
Sample source location	Vancouver, BC, Canada
Keywords	Gene expression, prenatal alcohol, adjuvant, arthritis, rat

Direct link to deposited data

http://www.ncbi.nlm.nih.gov/geo/query/acc.cgi?acc=GSE63561

## 1 Experimental design, materials and methods

### 1.1 Experimental design

**Fig. 1:**
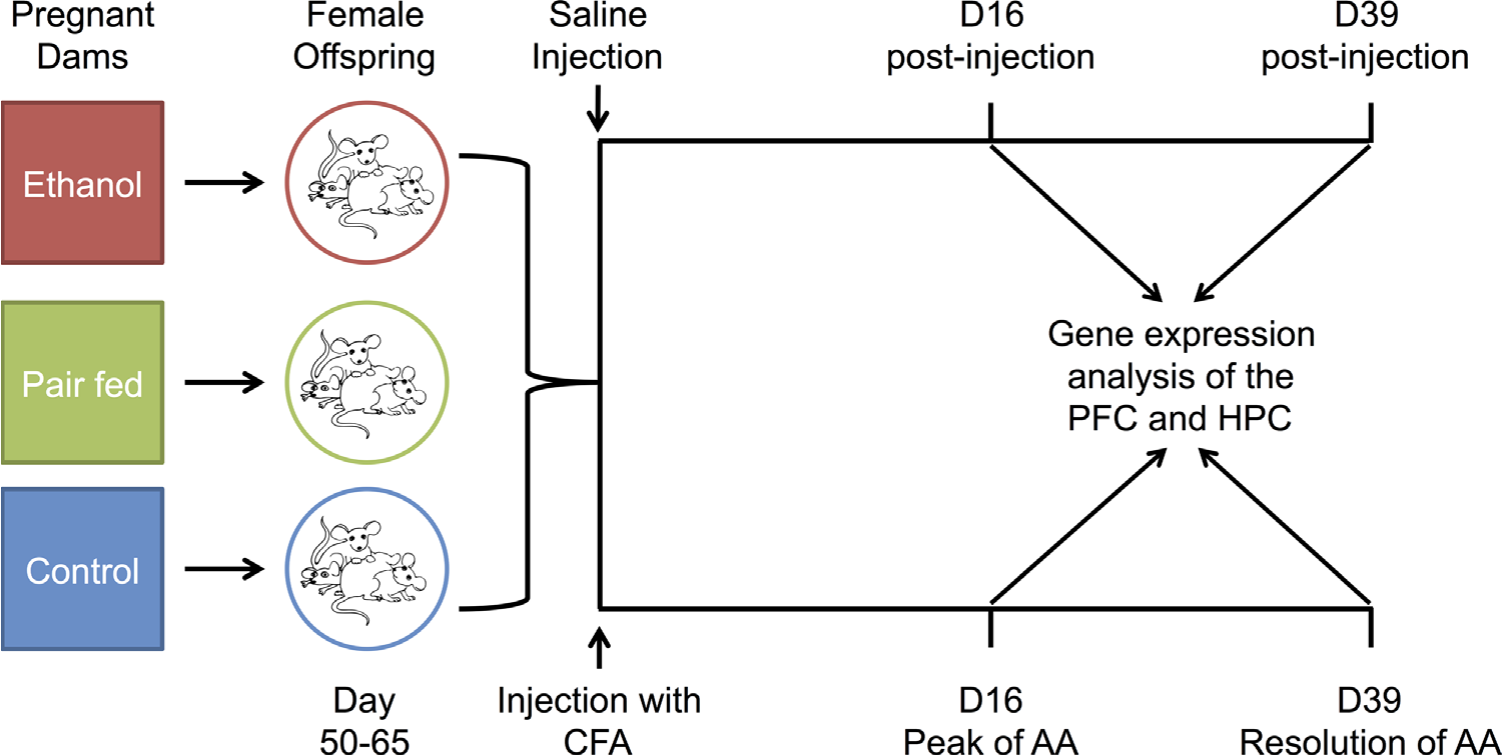
Overview of the experimental design prior to sample collection and microarray analysis. Adult female rats from one of three prenatal treatment groups, control, pair-fed, and prenatal alcohol exposed, were injected with complete Freund׳s adjuvant (CFA) to cause adjuvant-induced arthritis (AA). Animals were terminated 16 or 39 days post-injection and microarray analysis of gene expression was performed on the prefrontal cortex (PFC) and hippocampus (HPC).

Fetal alcohol spectrum disorder (FASD) currently affects 2–5% of children in North America, making in utero alcohol exposure one of the leading causes of neurodevelopmental disorder [9]. Prenatal alcohol exposure (PAE) adversely alters the development of the immune system, increasing the organism׳s susceptibility to immune and inflammatory challenges throughout life. Utilizing an adjuvant-induced arthritis (AA) paradigm, we have previously shown that PAE increases the course and severity of arthritis in female rats [14]. However, the molecular mechanisms underlying this vulnerability are not fully understood. As the prefrontal cortex (PFC) and hippocampus (HPC) are involved in neuroimmune function, alterations to their gene expression profile could result in abnormal steady-state functions and response to immune challenges. To test this hypothesis, we investigated the long-term effects of PAE on gene expression in the adult rat brain, resulting in the identification PAE-specific changes under both steady-state and challenge conditions [8]. These data were deposited into the Gene Expression Omnibus (GEO) Series GSE63561, which contains 192 gene expression microarray samples, and includes both technical replicates and analyzed samples. These samples were generated from adult female Sprague–Dawley rats from three prenatal treatment groups: ad libitum-fed control (C), pairfed (PF), and prenatal alcohol exposed (PAE). Animals were injected with either saline or complete Freund׳s adjuvant (CFA) to induce arthritis, and terminated at the peak of inflammation or during the recovery phase of arthritis (16 days or 39 days post-injection, respectively). The prefrontal cortex (PFC) and hippocampus (HPC) were dissected from whole frozen brains and gene expression data were obtained using the Illumina Rat-Ref12 expression microarray (Fig. 1). In saline-injected animals (steady-state conditions), we identified changes in gene expression and altered activation states of upstream regulators specific to PAE in both brain regions. At the peak of inflammation, we not only uncovered PAE-specific changes in gene expression, but also a failure of PAE animals to mount an appropriate response to the inflammatory challenge [8].

### 1.2 Breeding and prenatal alcohol exposure

All animal protocols were approved by the University of British Columbia Animal Care Committee and are consistent with the NIH Guide for the Care and Use of Laboratory Animals [10]. Complete details of the breeding and handling procedures have been previously published [3]. Briefly, Sprague Dawley rats were obtained from the Animal Care Center at the University of British Columbia, and group-housed for 1–2 weeks prior to breeding, with ad libitum access to standard lab chow (Jamieson׳s Pet Food Distributors, Ltd., Delta, BC, Canada). Female and male animals were then co-housed in stainless steel cages with mesh fronts and floors, with wax paper under the cages, which was checked daily for the presence of vaginal plugs indicating gestation day (GD) 1. On GD 1, pregnant dams were singly housed, and assigned to one of three treatment groups – a control group (C; fed laboratory chow ad libitum), an ethanol-fed group (prenatal alcohol exposed (PAE); ad libitum access to liquid ethanol diet, with 36% calories derived from ethanol), or a pair-fed group (PF; liquid control diet, with maltose-dextrin isocalorically substituted for ethanol, in the amount consumed by an ethanol-consuming partner, matched for g/kg body weight/day of gestation). Experimental diets were administered from GD 1–21 (Weinberg/Kiever Ethanol Diet #710324, Weinberg/Kiever Control Diet #710109, Dyets Inc., Bethlehem, PA), and all animals had ad libitum access to water. On GD 21, experimental diets were replaced with standard laboratory chow. At birth, litters were weighed and culled to 5 males and 5 females, when possible. Following weaning on postnatal day 22, female offspring were group-housed by litter (2–3 rats per cage) until testing. Blood alcohol levels for dams in this paradigm usually average 120–150 mg/dl [12,5].

### 1.3 Induction of arthritis

In adulthood (55–65 days of age), female offspring from C, PF, and PAE prenatal groups were selected for the study; females were tested due to their increased susceptibility to arthritis [13]. CFA was prepared by grinding powdered Mycobacterium tuberculosis H37 Ra (Difco laboratory, Detroit, MI) using a mortar and pestle, and dissolving it in 1 mL of mineral oil (Difco laboratory, Detroit, MI). Animals in the adjuvant group were given a 100 µL intradermal injection of a 12 mg/mL CFA suspension at the base of tail to induce arthritis (AA). In parallel, animals in the saline control group were given a 100 µL intradermal injection of a saline solution (0.9%) at the base of tail. Following injection, all animals were singly housed and monitored for clinical signs of arthritis. On post-injection days 7, 10, and every other day thereafter, animals were lightly anesthetized with isofluorane and clinical scores were assigned based on severity of paw redness and swelling (Supplementary information; results previously published in [14]).

### 1.4 Termination of animals

Two cohorts of C, PF, and PAE females were run in overlapping subsets for the analysis of gene expression in order to capture gene expression changes associated with the progression of AA. The first cohort was terminated 16 days post-injection, at the peak of arthritis, and the second was terminated 39 days post-injection, during the resolution phase of arthritis. Each cohort contained 27 adjuvant-injected (*n*=9 per prenatal treatment group) and 15–18 saline-injected animals (*n*=5–6 per prenatal treatment group). At termination, animals were removed one by one from the colony room, exposed to carbon dioxide for 30 seconds, and quickly decapitated. Brains were rapidly removed, snap-frozen on dry ice, and stored at −80 °C.

### 1.5 Tissue dissection and RNA extraction

The prefrontal cortex (PFC) and hippocampus (HPC) were dissected using RNase-free technique on brains pre-thawed at −20 °C for 30 min and then thawed on ice for 20 min. Dissected tissues were placed in chilled RNAlater and stored at −20 °C until RNA extraction. Of note, tissues were dissected in three batches, which ranged from December 2007 to May 2010 (Supplementary information). RNA and DNA were simultaneously extracted from samples using Qiagen׳s All-Prep DNA/RNA Mini kit (QIAGEN Inc., Toronto, ON). Brain tissue was placed in lysis buffer and disrupted using a 20 G needle, followed by a round of mechanical sheering using a 23 G needle, and a final homogenization by QIAshredder columns (QIAGEN Inc., Toronto, ON). RNA and DNA were then extracted from lysates according to the manufacturer׳s instructions and the DNase digestion step was included in the process to remove DNA contamination that might confound gene expression data. One hippocampus from a PF female in the adjuvant-treated group terminated on day 39 was excluded at this stage due to contamination during RNA extraction. Samples were extracted in two batches, either in the fall of 2009 or summer of 2010.

**Fig. 2:**
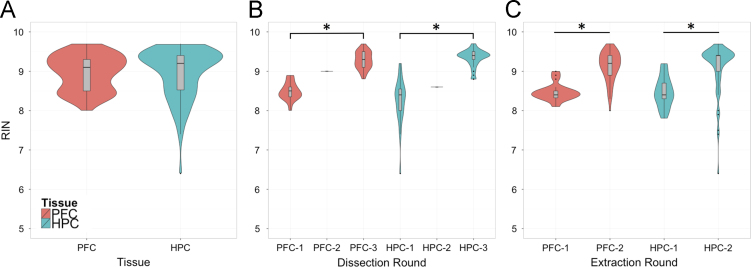
RNA integrity numbers (RIN) differ between extraction and dissection rounds. (A) No difference in average RIN was identified between samples from the prefrontal cortex (PFC) and the hippocampus (HPC). (B) Significant effects of tissue dissection round were identified between round 1 and round 3 in both tissues. Dissection round 2 contained a single sample in both tissues and was not included in statistical analysis. (C) Significant effects of RNA extraction round were also observed in both tissues. ^⁎^Denotes statistical significance below *p*<0.00001 by Welch׳s *t*-test.

RNA integrity numbers (RIN) were assayed using the Agilent BioAnalyzer mRNA Nano assay and no samples were excluded due to low RNA quality. The mean RIN was above 9 for both the PFC and HPC and there were no significant differences between tissues (Fig. 2A). The HPC showed more variability in the quality of RNA and a single sample skewed the distribution of this tissue, but remained above the minimal value of 6 (Supplementary Table 2). Certain samples from the HPC also produced intensities too low to accurately measure RIN, marked as NA, but were not excluded as they appeared of good quality upon visual inspection of the raw Bioanalyzer peaks (Supplementary information). Significant differences in RIN between extraction and dissection rounds were also observed in both tissues (Fig. 2B and C; *p*<1e−5, Welch׳s *t*-test), which were potentially due to slight procedural differences between the individuals performing these steps and/or longer storage of the first batch as dissected tissue prior to RNA extraction.

### 1.6 Microarray assay of whole genome gene expression

Total RNA (250 ng) was amplified into cRNA in batches of ~24 samples using the Ambion Illumina TotalPrep RNA Amplification kit (Life Technologies, Carlsbad, CA). Samples were randomly distributed across amplification cohorts to avoid confounding batches with experimental treatment groups (Supplementary information). One amplification replicate was included in the PFC set of samples to assay for reproducibility between amplification rounds. Expression data were obtained using the Illumina RatRef-12 Expression BeadChip microarray, which contains 12 arrays per chip and provides probe-level data for all expressed genes in the rat genome (~1 probe per gene). In concordance with the manufacturer׳s recommendations, 750 ng of cRNA from a single sample was hybridized to each array, and arrays were scanned on the Illumina iScan to obtain raw bead-level expression data. Experimental groups were counter-balanced across chips, such that chip batch was not confounded with experimental groups, as well as dissection, extraction, or amplification batch (Supplementary information). A total of 96 arrays were run for each tissue. In addition to the 87 experimental samples, PFC arrays also included 1 amplification replicate and 8 hybridization replicates. HPC arrays consisted of 84 experimental samples, as well as 12 hybridization replicates. Hybridization replicates in the PFC dataset originated from a single sample, while those in the HPC dataset originated from 4 different samples. Arrays for each tissue were run separately on different days, due to a processing limit of 8 chips, or 96 arrays. Of note, PFC samples were amplified in August 2010 and run in September 2010, while HPC samples were amplified in December 2010 and run in January 2011.

### 1.7 Data pre-processing and quality control

Datasets from both tissues were processed and analyzed separately throughout the entire study, as performing a comparison among prenatal treatment groups was more important than a direct comparison between tissues. Following the acquisition of raw expression data for each array, spatial artifacts were identified using the BASH algorithm [1] and masked prior to calculating the summarized expression values for each probe. The bioconductor package *beadarray* [2] was then used to collapse raw bead-level data into probe-level data, which was log 2-transformed for downstream applications.

**Fig. 3:**
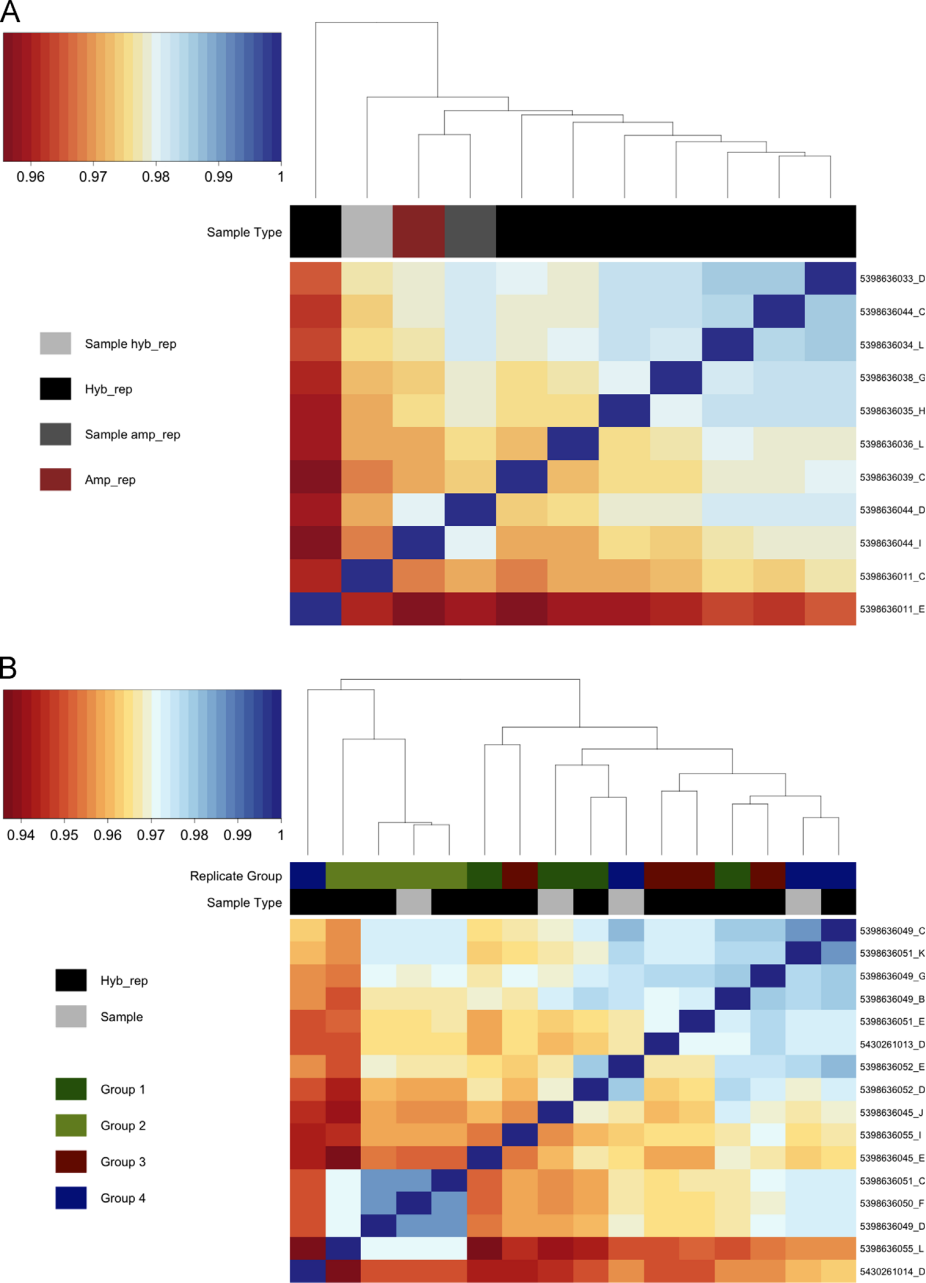
Technical replicates were highly correlated within the prefrontal cortex (PFC) and hippocampus (HPC). Quantile normalized hybridization and amplification replicates were compared by pairwise Pearson correlation and clustered according to inter-sample correlation values. (A) In the PFC, all hybridization replicates (Hyb_rep), consisting of 9 arrays from the same original sample, were highly correlated, with correlation values >0.95. The amplification replicates (Amp_rep), consisting of 2 arrays from the same sample, were also highly correlated and clustered together. (B) In the HPC, all hybridization replicates, which originated from 4 different samples (group), were highly correlated, with correlation values >0.94, and partially clustered by original sample group. Unique array identification numbers are displayed on the right side of each heatmap.

**Fig. 4:**
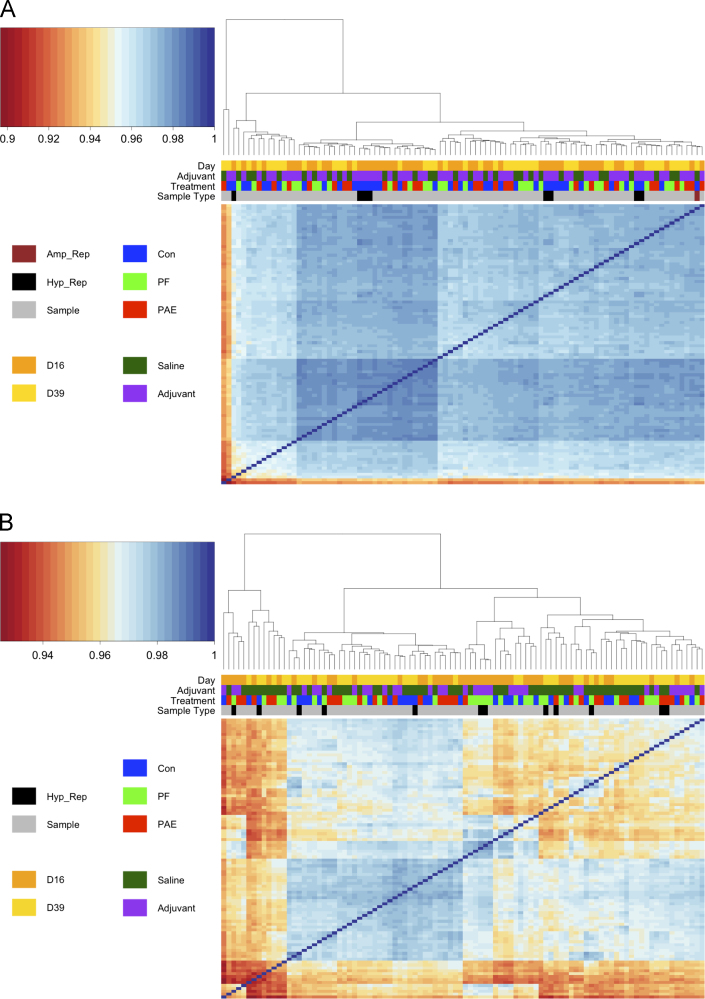
Most arrays were highly correlated within the prefrontal cortex (PFC) and hippocampus (HPC). Quantile normalized arrays were compared by pairwise Pearson correlation and clustered according to inter-sample correlation values. (A) In the PFC, most arrays were highly correlated, with correlation values >0.94. Two samples were identified as outliers, due to their separate clustering and apparent discordance with other arrays. (B) In the HPC, arrays were highly correlated, with correlation values >0.92, and no samples clustered individually. Samples in either tissue did not cluster by termination day (Day), adjuvant exposure (Adjuvant), prenatal treatment (Treatment), or sample type (Type).

Datasets were quantile-normalized in the *beadarray* package and pairwise Pearson correlations were calculated to compare expression profile correlations (Table 1). Hybridization replicates for both the PFC and HPC were highly correlated (>0.94), as were the amplification replicates in the PFC (>0.98) (Fig. 3), suggesting that microarray assays were carried out reproducibly. Although most samples within the same tissue were very highly correlated (Fig. 4), two samples from the PFC were identified as outliers, as their correlation values were considerably lower than those of their counterparts (Fig. 4A). These arrays, 5398636033_K and 5398636033_L, corresponded to a PAE sample from the day 39 saline group and a C sample from the day 39 adjuvant group respectively and contained large spatial artifacts. While outlier beads were masked during preprocessing and omitted when calculating expression levels, these extensive artifacts likely contributed to the observed discrepancy in sample correlation. As such, these two samples were removed from subsequent analyses. No samples were flagged as outliers in the HPC dataset (Fig. 4B), as all arrays showed correlation values >0.92.

**Fig. 5:**
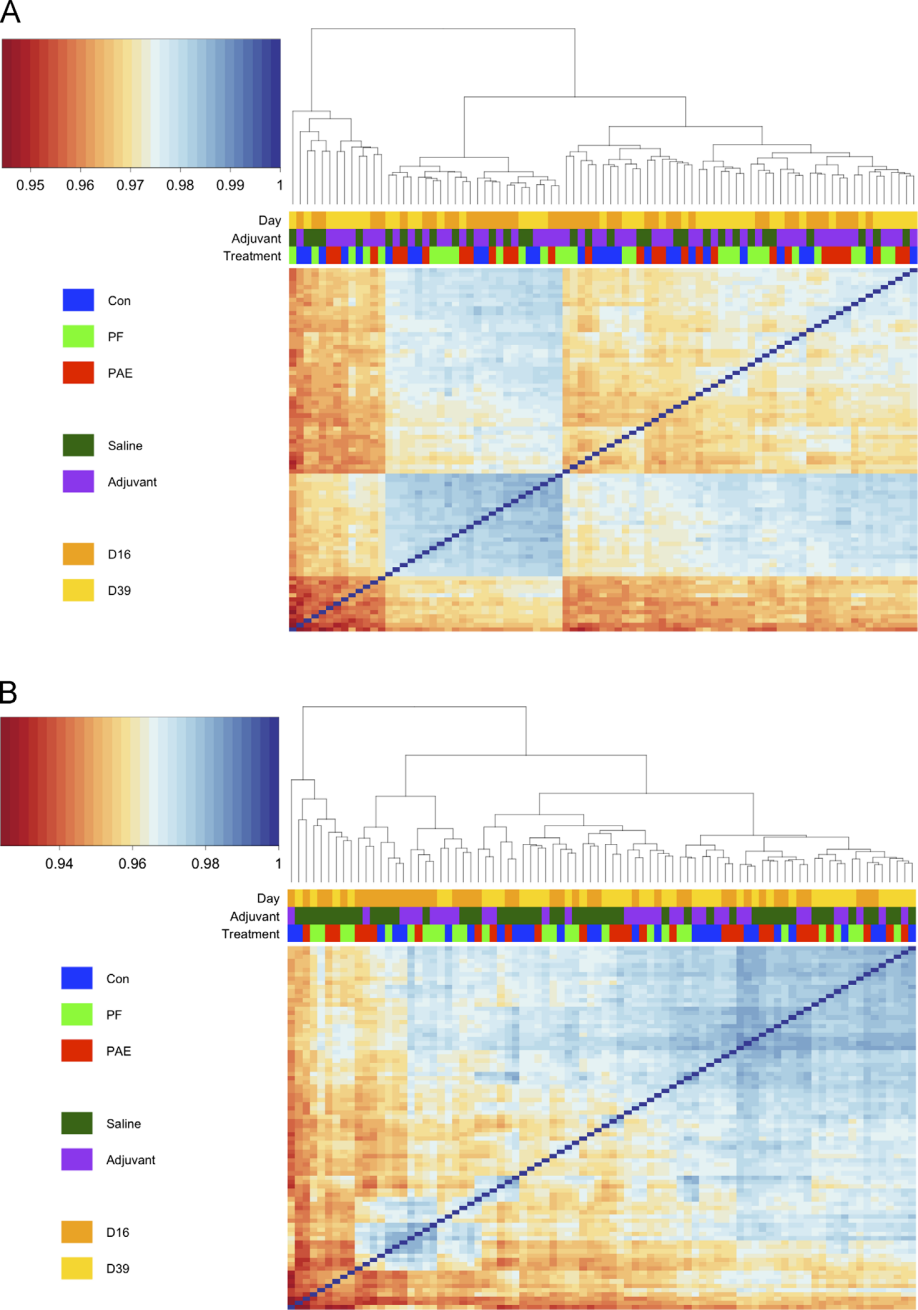
Filtered and quantile normalized samples were highly correlated in the prefrontal cortex (PFC) and hippocampus (HPC). Arrays were filtered to remove control and low-quality probes, then quantile normalized prior to pairwise Pearson correlation and clustering according to inter-sample correlation values. (A) In the PFC, all samples were highly correlated, with values >0.95. Samples partially clustered by termination day (Day) and adjuvant exposure (Adjuvant), but did not reflect any effect of prenatal treatment (Treatment). (B) In the HPC, all samples were also highly correlated, with values >0.95. Samples partially clustered by termination day and adjuvant exposure, but did not cluster by prenatal treatment.

Outliers and technical replicates were removed from the datasets for gene expression analyses. The final dataset for each tissue consisted of 85 samples in the PFC and 84 samples in the HPC (Table 2). The original probe-level expression data, prior to quantile normalization, was filtered to remove control probes and those with a detection *p*-value >0.05 compared to negative controls. After filtering, 20,215 probes remained in the PFC dataset, and 20,069 probes remained in the HPC dataset, from a total 23,350 probes. The filtered, log 2-transformed gene expression profiles were then quantile-normalized across arrays within each tissue. No samples stood out as outliers at this stage, as per Pearson correlations (Fig. 5).

## 2 Data analysis summary

### 2.1 Exploratory data analysis

**Fig. 6:**
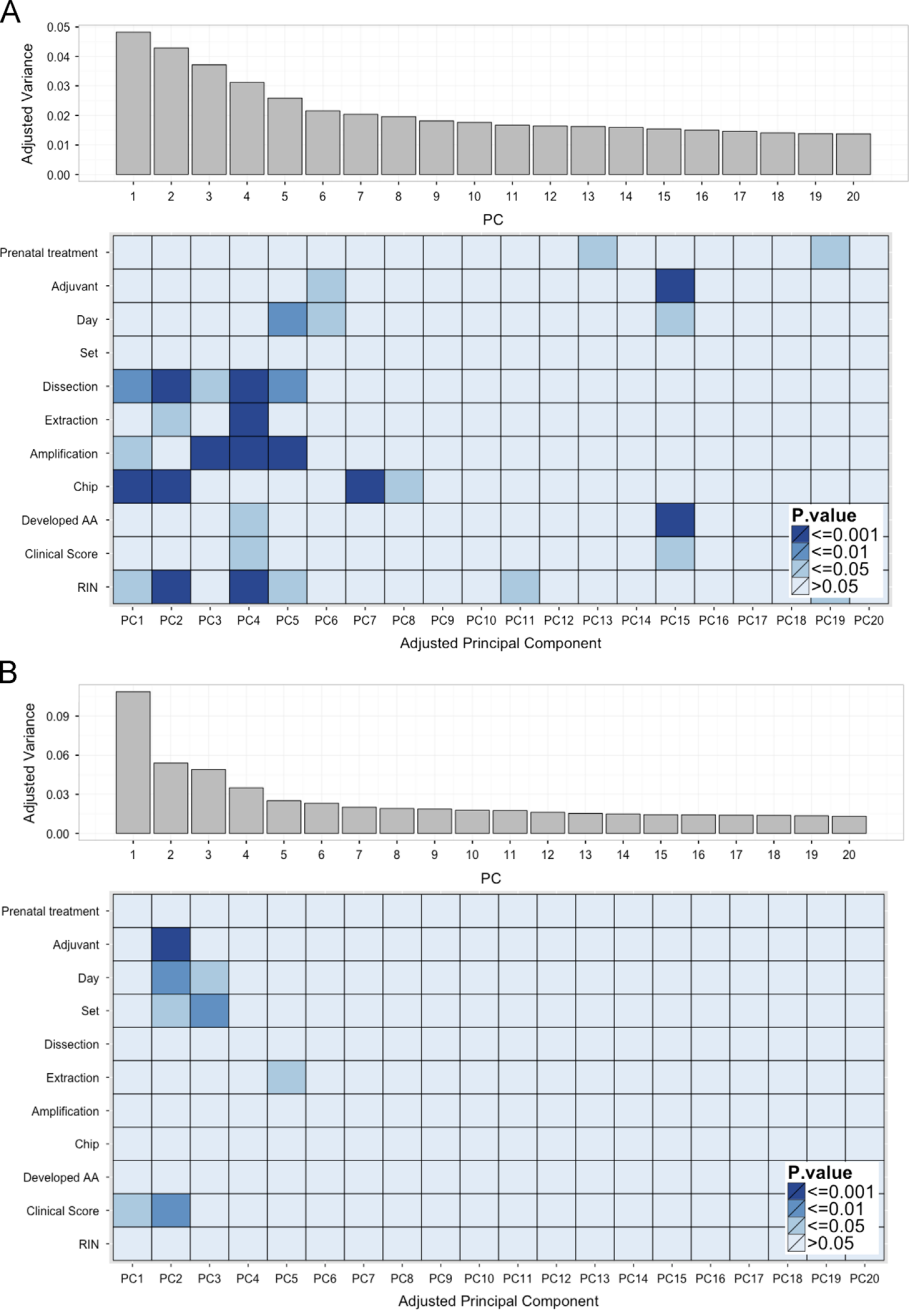
Principal component analysis of normalized and filtered datasets from the prefrontal cortex (PFC) and hippocampus (HPC) identified significant batch effects. Adjusted principal components (PC) were analyzed to identify associations with metadata variables and visualize significant sources of variation within the data. (A) In the PFC, batch effects, such dissection, extraction, and amplification round, as well as inter-chip variation, and RNA integrity numbers (RIN) accounted for a large proportion of the adjusted variance. Treatment effects, such as termination day (Day), adjuvant treatment (Adjuvant), total clinical score (Clinical Score), and prenatal treatment (Treatment) accounted for some variance in later principal components. (B) In the HPC, effects from the experimental batch (Set) and extraction round were identified in the first principal components. Effects of termination day, adjuvant exposure, and total clinical score also accounted for a significant proportion of the variance.

Upon visual examination of sample clustering in the heatmaps, the samples of the PFC and HPC showed a slight tendency to cluster by adjuvant exposure and stage of arthritis (day 16, peak of inflammation, or day 39, recovery phase), but not by prenatal treatment (Fig. 5). However, sample clustering also partially reflected batch effects from the different extraction, dissection, and amplification rounds, as well as variation between chips (data not shown). Thus, principal component analysis (PCA) was used to further investigate the expression heterogeneity caused by these external covariates (Fig. 6). In order to visualize more subtle variation within the data, the first principal component was omitted, as it did not correlate with any metadata variables and represented a very large proportion of the variance, masking other significant effects. The remaining components were adjusted based on the variance of the first principal component and analyzed to identify associations with different metadata variables and obtain a visual representation of variation sources within the datasets.

In the PFC, the majority of variation was correlated with batch effects from the different dissection, extraction, and amplification rounds, as well as chip-specific effects (Fig. 6A). RIN values also appeared to play a significant role in sample variation, but this effect may be confounded by the significant differences between dissection and extraction rounds (Fig. 2). Slight effects of experimental treatments, such as stage of arthritis (day 16 or 39), adjuvant exposure, and prenatal treatment, were also observed in later principal components. The major contributors to these effects were stage of arthritis and clinical score, which is closely related to the response to adjuvant treatment. However, the low proportion of the variance represented by experimental treatments suggests that their effects on gene expression are subtle, which was taken into consideration in downstream analyses.

By contrast, considerably fewer batch effects were observed in the HPC than in the PFC, as only batches of animals terminated together (set) and extraction round were correlated with the first five principal components. This may have been due to improved technique during RNA extraction, as HPC samples were processed after the PFC samples, or more consistent HPC dissections thanks to its more distinct anatomical landmarks, which resulted in less technical and biological variability. Moreover, significant effects of stage of arthritis (day 16 or 39) and adjuvant treatment, including the related clinical score, were present in the HPC dataset, suggesting that this region is more susceptible to the effects of adjuvant on gene expression than the PFC. No effects of prenatal treatment were apparent in this exploratory analysis of hippocampal gene expression.

Of note, termination of animals in this study occurred over several months due to the large number of animals, frequent monitoring throughout AA, and extensive processing involved at termination. While animals terminated together (sets) were counterbalanced for prenatal group and adjuvant treatment, they were not counterbalanced for stage of arthritis (day 16 or 39), and more adjuvant-injected (*n*=9 per prenatal treatment group) than saline-injected (*n*=5–6 per prenatal treatment group) animals were present in each cohort. As such, it is not completely unexpected that some variability in the data is associated with set. However, as animals were terminated in overlapping cohorts based on stage of arthritis, the effects of set observed by PCA were potentially confounded with termination day (i.e. peak of inflammation or recovery phase), or adjuvant treatment, which are also present in the first principal components. Furthermore, a large number of principal components did not correlate with any metadata variables in both tissues. These uncorrelated components may represent variation within the data caused by unknown factors, such as inter-individual differences and cell type composition, which could play a role in gene expression differences between groups.

### 2.2 Differential expression analysis

In order to correct for these unknown variables, as well as significant batch effects in the two datasets, surrogate variable analysis (*sva)* was used to generate variables representative of expression heterogeneity within the two datasets [7]. This method identifies eigenvectors of variation not associated with primary variables, which can then be incorporated as covariates during linear modeling to remove unwanted sources of heterogeneity. These surrogate variables were generated separately for animals in the day 16 and 39 cohorts in both the PFC and HPC, and the data were analyzed in two different ways, consistent with later bioinformatic analyses of gene expression changes (Table 3, Simplified_Rscript). In the first analysis, prenatal treatment was selected as the sole primary variable in order to test for steady-state gene expression in saline-injected animals. In the second analysis, prenatal treatment and adjuvant treatment were selected as primary variables in order to test differences in response to an inflammatory challenge compared to the response to saline in animals from the three prenatal treatment groups.

Surrogate variables generated by *sva* were included as covariates in linear modeling of gene expression using the *limma* package in the statistical program R [11]. Just as in surrogate variable analysis, gene expression changes were modeled in two different ways, with animals from day 16 and 39 separate from each other. First, the effects of prenatal treatment alone in saline-injected animals were analyzed to identify gene expression specific to the steady-state condition. Second, the interaction of prenatal treatment and adjuvant exposure in saline- versus adjuvant-injected animals was analyzed to identify differential responses to adjuvant exposure in each prenatal treatment group. Cohorts of saline-injected PAE, PF, and C females were terminated in parallel with adjuvant-injected animals on days 16 and 39 post-injection. In all models, a moderated F-statistic was generated for each probe, which adjusted for multiple testing using Benjamini–Hochberg correction. As the effects of experimental treatments were subtle, the false-discovery rate (FDR) was controlled at 25% (*q*-value <0.25). Within probes below this cutoff, those with a *p*-value <0.05 in contrasts of interest were considered significant.

To identify PAE-specific changes in expression under steady-state conditions, genes were required to display differential expression in both PAE versus C and PAE versus PF contrasts, while showing no difference between PF and C animals (*p*>0.05). On day 16 post-injection, 15 differentially expressed probes specific to PAE animals were identified in the PFC, while 4 were identified in the HPC [8]. No significant changes were identified between prenatal treatment groups on day 39 post-injection. This absence of group effects may be due to greater intra-group variability caused by the frequent handling over the paradigm׳s 39 day period, or adaptation of the animals to the handling procedures. As such, the day 39 group was not included in the subsequent analyses.

To identify the specific response of PAE animals to adjuvant, gene expression differences were analyzed in two steps. First, gene expression differences between saline- and adjuvant-injected animals of the same prenatal treatment group were obtained. Next, lists of significant genes were contrasted to obtain those altered in PAE versus C and PF, while showing no difference between C and PF. In addition, significant gene expression changes in saline- versus adjuvant-treated animals present in both control groups, but absent in PAE animal were also considered unique PAE responses. On Day 16 post-injection, 8 probes in the PFC and 4 in the HPC were differentially expressed in PAE compared to PF and C animals, while all groups showed a global up-regulation of mRNA levels [8]. Please refer to the attached *Simplified_Rscript* for an abridged version of these analyses.

As the majority of probes on the RatRef-12 beadchip were designed based on transcripts in RefSeq with only provisional annotation, the sequences for significant probes were queried against the most recent RefSeq database for *Rattus norvegicus* to establish the identity of target transcripts. Gene Ontology was performed on all genes using the gene-score resampling method in ermineJ [6], and Ingenuity^©^ Upstream Regulator Analysis (URA, Ingenuity Systems Inc., Redwood City, CA) was performed on genes with a fold change ≥1.2 and *p-value*<0.05 between treatments in order to identify effects of PAE and adjuvant injection on the transcriptome [8].

## 3 Conclusion

The exploratory portion of our analysis stresses the importance of consistent procedures, as well as balanced groups during all phases of studies requiring staggered experiments with large group sizes. Balanced experiments could mitigate the impact of confounding factors, facilitating their removal through bioinformatic methods such as ComBat [4], or by including them as covariates during linear modeling. Importantly, in the present study, despite some increase in variability due to the necessity to run cohorts in overlapping sets, significant effects of prenatal treatment were observed, suggesting relatively robust effects of prenatal alcohol exposure on steady-state functions and the response to an inflammatory challenge.

### 3.1 GEO submission

Probe-level data for this study were deposited on the GEO (GSE63561), along with metadata describing experimental treatment groups. As described, spatial artifacts were masked from the dataset and data were log 2-transformed prior to submission of raw and quantile-normalized data. Moreover, due to GEO submission requirements, the datasets were filtered to include only probes associated with a gene. As such, the current GEO files do not contain any control probes, although these additional data are available upon request.

## Figures and Tables

**Table 1:** Quantile distributions of pairwise Pearson correlations of arrays from the prefrontal cortex (PFC) and hippocampus (HPC).

**Tissue**	**Group**	***n***	**0%**	**25%**	**50%**	**75%**	**100%**
PFC	All samples	96	0.897	0.966	0.971	0.976	1.000
	Technical replicates	11	0.956	0.972	0.978	0.982	1.000
	Amplification replicates	2	0.980	0.980	0.990	1.000	1.000
	Hybridization replicates	9	0.957	0.971	0.979	0.982	1.000
	Samples only – no outliers	85	0.944	0.968	0.972	0.977	1.000
HPC	All samples	96	0.925	0.956	0.965	0.971	1.000
	Hybridization replicates	16	0.936	0.958	0.966	0.973	1.000
	Hybridization Group 1	4	0.961	0.968	0.971	0.982	1.000
	Hybridization Group 2	4	0.971	0.972	0.986	0.990	1.000
	Hybridization Group 3	4	0.964	0.969	0.974	0.984	1.000
	Hybridization Group 4	4	0.957	0.963	0.980	0.989	1.000
	Samples only – no outliers	84	0.924	0.958	0.967	0.972	1.000

**Table 2:** Distribution of sample numbers between different prenatal treatments, termination days, and adjuvant-induced arthritis (AA) treatments for the prefrontal cortex (PFC) and hippocampus (HPC).

**Prenatal treatment**	**Termination day**	**AA treatment**	**PFC**	**HPC**
Control	Day 16	Saline	5	5
		Adjuvant	9	9
	Day 39	Saline	6	6
		Adjuvant	8	8
Pair-fed	Day 16	Saline	5	5
		Adjuvant	9	9
	Day 39	Saline	6	6
		Adjuvant	9	7
Prenatal alcohol exposure	Day 16	Saline	5	5
		Adjuvant	9	9
	Day 39	Saline	5	6
		Adjuvant	9	9
		**Total**	**85**	**84**

**Table 3:** Total number of variables identified by surrogate variable analysis between steady-state and saline versus adjuvant in the prefrontal cortex (PFC) and hippocampus (HPC).

**Analysis**	**Termination day**	**PFC**	**HPC**
Steady-state (prenatal treatment)	Day 16	6	2
	Day 39	4	6
Saline versus adjuvant (prenatal treatment ⁎ adjuvant exposure)	Day 16	15	8
	Day 39	11	12

## Footnotes

Appendix A Supplementary data associated with this article can be found in the online version at doi:10.1016/j.dib.2015.05.007.
